# Diversity of Fungal DNA Methyltransferases and Their Association With DNA Methylation Patterns

**DOI:** 10.3389/fmicb.2020.616922

**Published:** 2021-01-22

**Authors:** Yu-Shin Nai, Yu-Chun Huang, Ming-Ren Yen, Pao-Yang Chen

**Affiliations:** ^1^Department of Entomology, National Chung Hsing University, Taichung, Taiwan; ^2^Institute of Plant and Microbial Biology, Academia Sinica, Taipei, Taiwan; ^3^Bioinformatics Program, Taiwan International Graduate Program, National Taiwan University, Taipei, Taiwan; ^4^Bioinformatics Program, Institute of Information Science, Taiwan International Graduate Program, Academia Sinica, Taipei, Taiwan

**Keywords:** fungi, epigenomics, DNA methyltransferases, DNA methylation, DNMT5, transposable element

## Abstract

DNA methyltransferases (DNMTs) are a group of proteins that catalyze DNA methylation by transferring a methyl group to DNA. The genetic variation in DNMTs results in differential DNA methylation patterns associated with various biological processes. In fungal species, DNMTs and their DNA methylation profiles were found to be very diverse and have gained many research interests. We reviewed fungal DNMTs in terms of their biological functions, protein domain structures, and their associated epigenetic regulations compared to those known in plant and animal systems. In addition, we summarized recent reports on potential RNA-directed DNA methylation (RdDM) related to DNMT5 in fungi. We surveyed up to 40 fungal species with published genome-wide DNA methylation profiles (methylomes) and presented the associations between the specific patterns of fungal DNA methylation and their DNMTs based on a phylogenetic tree of protein domain structures. For example, the main DNMTs in Basidiomycota, DNMT1 with RFD domain + DNMT5, contributing to CG methylation preference, were distinct from RID + Dim-2 in Ascomycota, resulting in a non-CG methylation preference. Lastly, we revealed that the dynamic methylation involved in fungal life stage changes was particularly low in mycelium and DNA methylation was preferentially located in transposable elements (TEs). This review comprehensively discussed fungal DNMTs and methylomes and their connection with fungal development and taxonomy to present the diverse usages of DNA methylation in fungal genomes.

## Introduction

Epigenetic modifications are external modifications that do not change DNA sequences and play a role in regulating gene expression, among other important biological functions (Holliday, 2006). The major epigenetic mechanisms across the kingdoms of animals, plants, and fungi include DNA methylation, histone modifications, chromatin remodeling, and small non-coding RNAs (Jeltsch, 2010). In this review, we focused on DNA methylation—specifically the 5-methylcytosines (5mC). They are more studied to date comparing with the other types of DNA modifications, such as N4-methylcytosine (4mC) and N6-methyladenine (6mA), and may influence the development of fungal species.

Genome-wide DNA methylation can now be determined at single-base resolution by whole-genome bisulfite sequencing (WGBS) (Su et al., 2011). Treatment of DNA with sodium bisulfite converts unmethylated cytosines (C) to uracils (U); thereafter, PCR amplification will convert these Us to thymines (T), while the methylated cytosines are protected and thus not converted. By using WGBS, researchers have comprehensively compared DNA methylation in living organisms. With the increase in whole-genome sequence data, we now have an unprecedented opportunity for obtaining complete genome-wide DNA methylation maps (DNA methylomes) (Zemach et al., 2010).

Comparison of DNA methylomes across more than 20 eukaryote genomes (including those of animals, plants and fungi) has revealed that most of the genomes undergo DNA methylation, while only a few eukaryotes show zero or little evidence of DNA methylation, i.e., yeast (*Saccharomyces cerevisiae)*, fruit fly (*Drosophila melanogaster*) and roundworm (*Caenorhabditis elegans*) (Jeltsch, 2010). DNA methylation was also detected in some model fungal species (Filippovich et al., 2004; Mishra et al., 2011; Montanini et al., 2014). For example, DNA methylation in *Neurospora crassa* (red bread mold; Ascomycota) is crucial for balancing the formation of sexual and asexual reproductive structures (Filippovich et al., 2004). In the human fungal pathogen *Candida albicans*, DNA methylation has been recently reported to be primarily located within structural genes and associated with the dimorphic transition between yeast and hyphal forms, switching between white and opaque cells, and iron metabolism (Mishra et al., 2011). In black truffle, *Tuber melanosporum*, the DNA methylation pattern revealed selective targeting of transposable elements (TEs) rather than gene bodies, and demethylation treatment changed its phenotype (Montanini et al., 2014). Altogether, the current evidence supports the importance of fungal DNA methylation associated with changes in reproductive structures, the dimorphic transition and phenotypes.

In this review, we start by discussing the characteristics and functions of all DNA methyltransferases (DNMTs), which transfer a methyl group to the C-5 position of the cytosine ring and establish DNA methylation, especially fungal-specific DNMT5- and DNMT5-associated potential RNA-directed DNA methylation (RdDM). To explore the correlations between fungal DNMTs and DNA methylation, we list the DNMTs and summarize the DNA methylation data from 40 fungal genomes, mainly belonging to Ascomycota and Basidiomycota, as well as Mucoromycota and Myxomycota (Table 1). In addition, we perform a comprehensive survey of DNMT phylogenies, domain structures, and fungal DNA methylation levels at different life stages. A conserved phylogeny based on the fungal DNMT1 family and Masc1/RID (repeat-induced point mutation defective) is also described in this review. Although the DNA methylation levels in different fungal species were diverse, the dynamics of methylation changed during the fungal life cycle, and a preference for methylated locations in fungi was found. Our review presents a summary of a large survey of fungal DNA methylation levels and the preference for DNMTs in specific groups of fungi.

**TABLE 1:** List of 40 fungi with the descriptions of methylation data.

**Division**	**Subphylum**	**Order**	**Organism**	**Method**	**Stage**	**ave. 5mC**	**mCG**	**mCHG**	**mCHH**	**Mappability (%)**	**References**
Basidomycota	Agaricomycotina	Agaricales	*Coprinopsis cinerea*	WGBS	Mycelia	3.03%	7.8%	0.6%	0.6%	89	Zemach et al., 2010
			*Laccaria bicolor*	WGBS	Mycelia	3.73%	10.1%	0.5%	0.6%	57	Zemach et al., 2010
			*Agaricus bisporus*	HPLC	Mycelia; yeast-like cells	4.01%	NA	NA	NA	NA	Binz et al., 1998
			*Schizophyllum commune*	MSRE	Mycelia	NA	NA	NA	NA	NA	Specht et al., 1984; Buckner et al., 1988
			*Armillaria bulbosa*	HPLC	Mycelia; yeast-like cells	4.34%	NA	NA	NA	NA	Binz et al., 1998
			*Pleurotus eryngii* Subsp. *Tuoliensis*	MSRE-PCR	Mycelia	NA	NA	NA	NA	NA	Hua et al., 2017
			*Pleurotus eryngii var. eryngii*	WGBS	Mycelia	NA	16.40%	1.30%	1.50%	88.6	Zhang et al., 2018
			*Pleurotus tuoliensis*	WGBS	Mycelia	NA	14.60%	1.40%	1.70%	86.7	Zhang et al., 2018
			*Pleurotus ostreatus*	WGBS	Mycelia PC 15 (*n*)	NA	2.76%	0.38%	0.45%	72	Borgognone et al., 2018
					Mycelia PC 9 (*n*)	NA	4.37%	0.62%	0.84%	59	
					Mycelia N001-HyB (*n* + *n*)	NA	3.96%	0.51%	0.65%	61	
					Mycelia M_N001 (*n* + *n*)	NA	6.48%	0.45%	0.59%	64	
					Primordia	NA	6.69%	0.43%	0.54%	59	
					Mature fruitbodies	NA	6.54%	0.40%	0.51%	63	
		Russulales	*Heterobasidion parviporum*	WGBS	Conidiospores (SPORE)	3.93%	8.43%	2.40%	2.20%	87.02	Zeng et al., 2019
					Mycelia (MYCEL)	3.53%	7.23%	2.47%	2.10%	88.36	
					Saprotrophic growth (SAP)	4.33%	8.03%	3.10%	3.00%	88.18	
					Necrotrophic growth (NECT)	4.57%	8.30%	3.27%	3.17%	88.65	
		Polyporales	*Postia placenta*	WGBS	Mycelia	3.10%	4.9%	1.2%	1.0%	80	Zemach et al., 2010
			*Ganoderma sinense*	WGBS	Mycelia	4.64%	10.2%	0.8%	1.0%	77	Zhu et al., 2015
			*Sporotrichum dimorphosporum*	RE-NNA	Mycelia	0.20%	NA	NA	NA	NA	Antequera et al., 1984
	Ustilaginomycotina	Ustilaginales	*Ustilago maydis*	HPLC	Mycelia; yeast-like cells	2.26%	NA	NA	NA	NA	Binz et al., 1998
			*Ustilago violaceae*	HPLC	Mycelia; yeast-like cells	2.14%	NA	NA	NA	NA	Binz et al., 1998
Ascomycota	Pezizomycotina	Diaporthales	*Cryphonectria parasitica*	WGBS	Mycelia	3.90%	0.09%	4.30%	4.30%	71.9	So et al., 2018
		Eurotiales	*Aspergillus flavus*	WGBS	Mycelia	–	NA	NA	NA	NA	Liu et al., 2012
		Magnaporthales	*Magnaporthe oryzae*	WGBS	Conidia	0.613%	0.6%	0.6%	0.6%	83	Jeon et al., 2015
					Mycelia	0.554%	0.6%	0.5%	0.5%	82	
					Appressoria	0.553%	0.5%	0.5%	0.6%	79	
		Sordariales	*Neurospora crassa*	GC/MS	Conidia	0.36%	NA	NA	NA	NA	Russell et al., 1987
					Conidial germlings	0.40%	NA	NA	NA	NA	
					Early exponential mycelia	0.24%	NA	NA	NA	NA	
					Mid-exponential mycelia	0.24%	NA	NA	NA	NA	
					Stationary-phase mycelia	0.40%	NA	NA	NA	NA	
			*Neurospora crassa*	WGBS	Vegetative^*a*^	2.19%	2.08%	2.05%	2.19%	80.05	Hosseini et al., 2020
					Pre-sexual^*b*^	2.86%	2.67%	2.44%	2.98%	77.35	
			*Neurospora sitophila*	WGBS	Vegetative	1.77%	1.67%	1.83%	1.72%	80.5	
					Pre-sexual	1.79%	1.68%	1.85%	1.75%	82.1	
			*Neurospora tetrasperma L1*	WGBS	Vegetative	1.28%	1.23%	1.59%	1.13%	82.325	
					Pre-sexual	1.41%	1.31%	1.67%	1.29%	80	
			*Neurospora tetrasperma L10*	WGBS	Vegetative	1.29%	1.30%	1.61%	1.13%	84.025	
					Pre-sexual	1.40%	1.37%	1.66%	1.28%	86.125	
			*Neurospora tetrasperma L6*	WGBS	Vegetative	1.21%	1.25%	1.59%	1.00%	77.05	
					Pre-sexual	1.24%	1.27%	1.63%	1.03%	76.775	
		Hypocreales	*Cordyceps militaris*	WGBS	Mycelia	0.48%	0.47%	0.48%	0.49%	NA	Xin et al., 2019
			*Fusarium oxysporum*	RE/Southern blot	Mycelia	NA	NA	NA	NA	NA	Kim, 1997
			*Metarhizium robertsii*	WGBS	Conidia 9d	0.403%	0.4%	0.4%	0.4%	93	Li et al., 2017
					Conidia 15d	0.394%	0.4%	0.4%	0.4%	93	
					Mycelia	0.392%	0.4%	0.4%	0.4%	91	
			*Metarhizium anisopliae*	WGBS	Mycelia	0.60%	0.40%	0.45%	1.85%	85.17	Sbaraini et al., 2019
		Onygenales	*Uncinocarpus reesii*	WGBS	Mycelia	0.68%	0.4%	1.0%	1.0%	94	Zemach et al., 2010
		Pezizales	*Tuber melanosporum*	WGBS	Fruitbody	12.823%	32.2%	11.6%	13.7%	79	Montanini et al., 2014
					Free-living mycelia	11.856%	32.4%	10.1%	13.0%	83	
			*Ascobolus immersus*	RE/Southern blot; WGBS	Mycelia	NA	NA	NA	NA	NA	Goyon et al., 1996
			*Phymatotrichum omnivorum*	HPLC	Mycelia; Dormant sclerotia	1.53%	NA	NA	NA	NA	Jupe et al., 1986
		*Pleosporales*	*Cochliobolus heterostrophus*	RE/Southern blot	Conidia	NA	NA	NA	NA	NA	Keller et al., 1991
		Ophiostomatales	*Ophiostoma novo-ulmi*	HPLC	Mycelia; yeast stage	1.38%	NA	NA	NA	NA	Binz et al., 1998
	Taphrinomycotina	Schizosaccharomycetales	*Schizosaccharomyces pombe*	LC-MS/MS	Yeast cell	-	NA	NA	NA	NA	Capuano et al., 2014
			*Yarrowia lipolytica*	GC/MS	Yeast cell	0.36%	NA	NA	NA	NA	Tang et al., 2012
	Saccharomycotina	Saccharomycetales	*Saccharomyces cerevisiae*	HPLC; LC-MS/MS	Yeast cell	-	NA	NA	NA	NA	Binz et al., 1998; Capuano et al., 2014
			*Candida albicans*	GC/MS	Mycelial-form cell; yeast-form cell	0.08%	NA	NA	NA	NA	Russell et al., 1987
Mucoromycota	Mucoromycotina	Mucorales	*Phycomyces blakesleeanus*	WGBS	Mycelia	0.94%	2.2%	0.3%	0.5%	60	Zemach et al., 2010
			*Phycomyces blakesleeanus*	RE-NNA	Mycelia	0.48%	NA	NA	NA	NA	Antequera et al., 1984, 1985
					Spore	2.90%	NA	NA	NA	NA	Antequera et al., 1984, 1985
			*Mucor rouxii*	RE; AFLP	Ungerminated spores, spherical (phase I) spores, phase II spores (germlings)	NA	NA	NA	NA	NA	Cano et al., 1988; Reyna-Lopez et al., 1997
Myxomycota		Physarales	*Physarum polycephalum*	HPLC	Spherules	5.42%	NA	NA	NA	NA	Fronk and Magiera, 1994

*WGBS, Whole Genome Bisulfite Sequencing; MSRE, Methylation-sensitive restriction enzyme; RE-NNA, Restriction enzyme and nearest-neighbor analysis; AFLP, Amplified Fragment Length Polymorphism; HPLC, High-performance liquid chromatography; GC/MS, Gas chromatography-mass spectrometry; LC-MS/MS, Liquid chromatograph/tandem mass spectrometer. –, Not-detectable; NA, Not available. ^*a*^Vegetative: mycelia, conidia. ^*b*^Pre-sexual: mycelia, conidia, and protoperithecia.*

### Five Major DNA Methyltransferases Have Been Characterized

DNMTs, containing a DNA methylase domain as the catalytic domain (PF00145), contribute to DNA methylation by transferring the activated methyl group (CH_3_) from S-adenosyl methionine (SAM) to the 5th position of a cytosine residue (5mC) (Law and Jacobsen, 2010). The DNA methylase domain consists of the catalytic core containing seven beta-sheet and three alpha-helices, and a target recognition domain (TRD) (Martin and McMillan, 2002). During the process of DNA methylation, the catalytic core and TRD are organized into a two-lobe architectural clef to harbor the DNA duplex to form the DNMT-DNA covalent complex in order to complete the enzyme activity (Yang and Xu, 2013).

**FIGURE 1:**
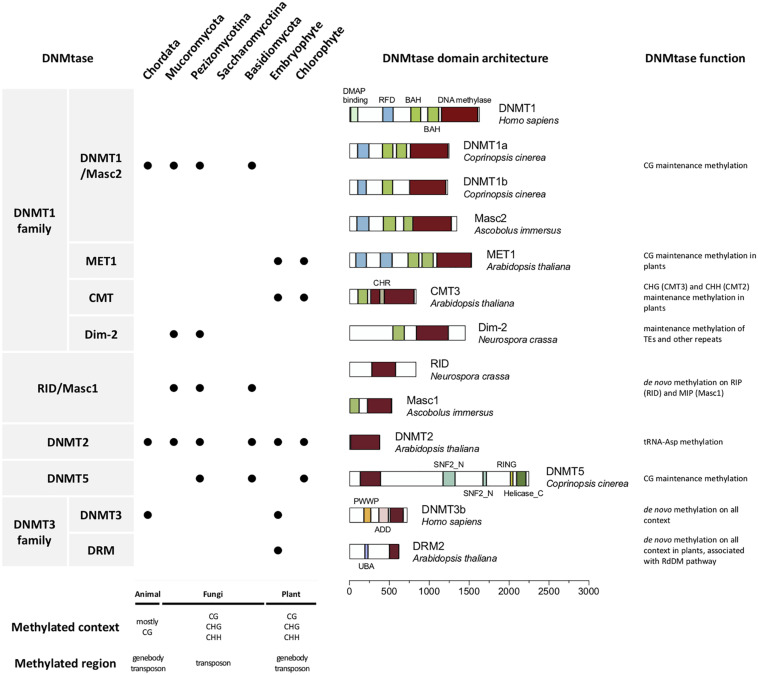
DNA methyltransferases in eukaryotes. The first column shows the names of DNMTs. The second column shows the presence or absence of DNMTs in the animal (Chordata), fungal (Mucoromycota, Pezizomicotina and Saccharomicotina of Ascomycota, and Basidiomycota), and plant (Embryophyte and Chlorophyte) kingdoms. The third column shows the domain architecture of the real DNMTs. The exact species used to generate these DNMT domain architectures are labeled. The fourth column shows function of the DNMTs. The methylation context and region often found in animals, fungi and plants are shown below.

DNMTs can be categorized into two types: those participating in “maintenance” and “*de novo*” methylation (Riggs, 1975). Maintenance DNMTs recognize hemimethylated DNA and copy the previous methylation patterns onto the nascent strands after each round of DNA replication, while *de novo* DNMTs methylate the unmethylated cytosines and establish novel DNA methylation patterns (Chen and Li, 2004). Different DNMTs have been characterized in prokaryotes and eukaryotes. Based on structural and functional similarity, DNMTs can be divided into five classes, including DNMT1, DNMT2, DNMT3/DRM, Masc1/RID, and DNMT5 (Figure 1; Ponger and Li, 2005; de Mendoza et al., 2020).

The DNMT1 subfamily, which is generally considered a key factor in maintenance methylation, contains animal and fungal DNMT1, as well as its homologous DNA methyltransferase 1 (MET1) in plants and Masc2 in *Ascobolus*, Dim-2 in Ascomycota, and plant-specific chromomethylases (CMTs) are also categorized as belonging to this subfamily, owing to having a domain structure and function similar to those of DNMT1 (Ponger and Li, 2005; Huang et al., 2016; de Mendoza et al., 2020).

The DNMT1 subfamily is known to have a bromo-adjacent homology (BAH) domain involved in transcriptional regulation such as gene silencing (Callebaut et al., 1999). The BAH domain is required for DNMT1 to target the replication foci during S phase, and links amongst DNA methylation, replication and transcriptional regulation (Yarychkivska et al., 2018). In plants, CMT3 binds H3K9me2 via both BAH and chromo domains, further suggesting that the BAH domain had the function of histone mark recognition (Du et al., 2012). In DNMT1 and MET1, the replication foci domain (RFD) is also observed, while the RFD is absent in the other two DNMT1 subgroups (Dim-2 and CMT). This RFD is responsible for targeting replication foci to discriminate between unmethylated and hemi-methylated DNA. This allows DNMT1 to methylate the correct residues (Syeda et al., 2011). It was also demonstrated that the RFD of DNMT1 functions as a transcriptional repressor that binds DMAP1 and HDAC2 at the replication foci during the S phase of mitosis (Rountree et al., 2000). Recently, the RFD domain of DNMT1 is referred to as a histone reader of K18/K23 ubiquitylated histone H3, in the assistance of DNMT1 to bind with H3-K18Ub/K23Ub. The module of Dnmt1 binding to two-mono-ubiquitylated H3 is required by Dnmt1 recruitment to the loci for DNA methylation, and the enhancement of its methyltransferase activity (Leonhardt et al., 1992; Ishiyama et al., 2017).

DNMT2 is another class of DNA methylase domain-containing enzymes that is highly conserved in eukaryotes and present in all kingdoms, including those of fungi, animals and plants (Huang et al., 2016). It is responsible for tRNA-Asp methylation and specifically methylates cytosine 38 in the anticodon loop. The function of DNMT2 is also conserved in different organisms, while restoration of human DNMT2 protein facilitated Dnmt2-deficient mouse, fly and Arabidopsis tRNA-Asp methylation *in vitro* (Goll et al., 2006). Although DNMT2 has zero or very low DNA methyltransferase activity, the DNA fragment in the structural context of a covalent DNA-tRNA hybrid can be more efficiently methylated than the all-ribo tRNAs (Kaiser et al., 2017).

DNMT3 is found in animals, and the homologous in plants are domains of rearranged methyltransferase (DRM) since they both play a role in *de novo* methylation. In mammals, DNMT3 contributes to the establishment of DNA methylation patterns during embryogenesis. In plants, DRM1/2 participates in the RNA-directed DNA methylation (RdDM) pathway guided by small RNAs (sRNAs) (Ehrlich et al., 1982; Jeltsch et al., 2006; Law and Jacobsen, 2010; Zemach et al., 2010; Matzke and Mosher, 2014). However, DNMT3 homologs are absent in fungal genomes and the genomes of some green algae (i.e., *Chlorella* sp. NC64A) and animal species (i.e., silkworm) (Zemach and Zilberman, 2010; Huang et al., 2016).

In fungi, the two fungi specific *de novo* DNMTs occurring on repeat sequences are Masc1 and RID. There is another DNMT, DNMT5, capable of fungal DNA methylation, which is also represented in some chlorophytes, stramenopiles, and haptophytes (Chen and Li, 2004; Huang et al., 2016; Bewick et al., 2019; Schmitz et al., 2019; de Mendoza et al., 2020). The details of fungal-specific DNMTs will be discussed in the following paragraphs.

## DNA Methyltransferases of Fungi

### DNMT1/Masc2, Dim-2, RID/Masc1, DNMT5, and DNMT2 Are Present in Fungi

Fungi are the principal decomposers in ecological systems. Based on their morphological, reproductive traits, and genetic differences, the fungal kingdom may be divided into nine major lineages under three groups of fungi species: (1) Zoosporic fungi (Opisthosporidia, Chytridiomycota, Neocallimastigomycota, Blastocladiomycota); (2) Zygomycetous fungi (Zoopagomycota, Mucoromycota, Glomeromycota); and (3) Dikarya (Ascomycota, Basidiomycota) (Naranjo-Ortiz and Gabaldón, 2019). Dikarya which in general produces dikaryons, is the largest and well-studied group of Fungi, presenting diverse lifestyles and phenotypes (Naranjo-Ortiz and Gabaldón, 2019). In this review we focus on the popular species of Ascomycota and Basidiomycota, and also few Mucoromycota and Myxomycota, depending on the availability of literatures and DNA methylation data.

Recently, the conserved configuration of fungal DNMTs was identified. Based on phylogenetic and domain architecture analyses of the DNMT sequences from fungal species, including Dikarya (Basidiomycota and Ascomycota) and early-diverging fungi (Blastocladiomycota, Chytridiomycota, and Mucoromycota), the fungal DNMTs can be grouped into two monophyletic superclades (Bewick et al., 2019). The first superclade is composed of DNMT1/Masc2, Dim-2, and RID/Masc1. The uncertain *de novo* DNMTX is also in this clade. The other superclade contains DNMT2 and Rad8/DNMT5. Based on evolutionary analysis (Bewick et al., 2019), Dim-2 existed in the other early-diverging fungi and Ascomycota, but was lost in Basidiomycota after the differentiation between Basidiomycota and Ascomycota in the subkingdom Dikarya. All fungi lack the *de novo* DNMT3 family, suggesting two independent gains in animals and plants or one single loss in the fungal ancestor that diverged from animals (Huang et al., 2016; Bewick et al., 2019). The distribution of DNMTs exhibited a common characteristic in which each fungus had at least one copy of the DNMT1 family, except for *Ustilago maydis*, *Malassezia globosa*, *Wallemia sebi* of Basidiomycota, and *Saccharomyces cerevisiae* from Saccharomycotina in Ascomycota (Huang et al., 2016).

Dim-2 is a DNMT1-related methyltransferase. Dim-2 is only found in fungi to mediate methylation of TEs and other repeats. In *Neurospora crassa*, the relationship between DNA methylation and histone lysine methylation is strong. Lysine 9 methylation of histone H3 (H3 mK9) is needed for DNA methylation mediated by Dim-2 (Goll and Bestor, 2005; Zemach et al., 2010). Moreover, it was proven that the modification state and sequence of DNA can affect the methylation states of accompanying histones in chromatin and *vice versa* (Goll and Bestor, 2005; Rose and Klose, 2014).

RID and Masc1, which are fungus-specific DNMTs that primarily function in genome defense mechanisms, are closely related to DNMT1 (Gladyshev, 2017). They are required for *de novo* methylation of TEs and repeats in both CG and non-CG contexts associated with repeat-induced point (RIP) and methylation-induced premeiotic (MIP) mutations, respectively (Chen and Li, 2004; Ponger and Li, 2005; Zemach et al., 2010; Freitag, 2017). The *masc1* gene is cloned from *Ascobolus immersus* and is required for the *de novo* methylation associated with MIP mutation, which is a process that scans the genome for DNA duplications and subsequently methylates the cytosines in these duplicated sequences (Malagnac et al., 1997). Moreover, the phenotype silencing is reversible during subsequent vegetative growth by the loss of cytosine methylation in MIP mutations (Goyon and Faugeron, 1989). The RIP mutation process in *N. crassa* is similar to the process of MIP mutation in *A. immerses*, and both RIP and MIP mutations act at a very precise stage of the sexual cycle (i.e., between fertilization and meiosis) (Selker et al., 1987; Grayburn and Selker, 1989). In contrast, the result of RIP mutation is irreversible; repeated DNA is detected in a pairwise manner and introduced with G-C to A-T transition mutations with a significant bias for 5’−CpA−3’ contexts, and most DNA methylation is found in relics of RIP mutations in *N. crassa* (Selker, 2002; Selker et al., 2003). In *Aspergillus nidulans*, DmtA is a DNMT homolog similar to RID mutation and Masc1 that contributes to sexual development, conidiation, sclerotial production, aflatoxin biosynthesis, and virulence (Lee et al., 2008; Yang et al., 2016).

DNMT5 has a unique architecture, including the RING finger with ubiquitination potential following the methyltransferase domain and a long C-terminal region of SNF2 family homology, which contains an ATP-dependent chromatin remodeling domain and helicase domain, suggesting that DNMT5 proteins are multifunctional enzymes (Ponger and Li, 2005; Huff and Zilberman, 2014; Dumesic et al., 2020). The complete CG methylation was lost in *Dnmt5* mutant *Cryptococcus neoformans*. As SNF2 domain ATP hydrolysis is required for driving CG methylation and hemimethylated DNA substrates preferentially activate SNF2 ATPase, DNMT5 is thought to maintain CG methylation with the help of SNF2 domain coupling the recognition and catalysis of hemimethylated DNA (Huff and Zilberman, 2014; Dumesic et al., 2020). DNMT5 is conserved in fungi, haptophytes, stramenopiles, and chlorophytes, indicating that it is ancestral in eukaryotes (de Mendoza et al., 2020). Another protein, the DNA repair protein Rad8, has high similarity to DNMT5 in terms of protein domain architecture. It has been recorded to have conserved RING finger-like motifs and SNF2-family helicase domains but without DNA methylase domains (Doe et al., 1993; Eisen et al., 1995; Ding and Forsburg, 2014); however, Huang and Zhang indicated that the fungal Rad8 subfamily contained DNA methylase domains and acted as DNA methyltransferases (Huang et al., 2016; Zhang et al., 2018). To clarify whether there are any misleading results, we compared the DNMT5 and Rad8 sequences of 20 fungal species reported in Bewick’s and Huang’s research (Huang et al., 2016; Bewick et al., 2019) and found that 18 of them were the same proteins, while the remaining 2 were homologs (blastp e-value ≤1e-30, mismatch <50 within 2,000 bp). Therefore, the Rad8 reported by Huang and Zhang is likely DNMT5, which is actually a DNMT.

In the ancestor of pathogenic *Cryptococcus* fungal species, *Kwoniella*, there is a putative DNA methyltransferase DNMTX containing a BAH domain and a DNA methylase domain and acting as a *de novo* methylase. DNMTX is considered an ancestrally lost DNMT in *Cryptococcus neoformans*, proven by the accumulation of 5mC when the gene for DNMTX was transferred into a *dnmt5* mutant of *C. neoformans* (Catania et al., 2020).

### Proteins With DNMT and SNF2 Family Domains May Be Involved in the RdDM-Like Pathway in Fungi

Helicase-like Snf2 superfamily proteins, such as CLSY1, DRD1 and SNF2-RING-HELICASE–LIKE-1 and -2 (FRG1 and FRG2), are required for RdDM in Arabidopsis (Groth et al., 2014). The chromatin remodeling-associated protein CLSY1 assists in the first step of the RdDM pathway, RNA polymerase IV (Pol IV)-dependent siRNA biogenesis, whereas DRD1 is involved in RNA polymerase V (Pol V)-mediated *de novo* methylation (Matzke and Mosher, 2014). Although the RdDM pathway is unique to plants, the product of the fungus-specific domain fusion event of the DNA methylase family and Snf2 family is suspected to act as DNMT (Huang et al., 2016). In *Pleurotus* fungi, the colocation of siRNA abundance and TE methylation also supports that methylation in TE regions is established by siRNA-directed DNA methylation. The genes encoding the proteins taking part in the RdDM pathway, including RNA-dependent RNA polymerase and Dicer-like and Argonaute proteins, are found in Basidiomycota fungi (Zhang et al., 2018). This evidence suggests that there might be an RdDM-like mechanism in the fungal kingdom. As DNMT5 contains the SNF2_N and helicase_C domains of the Snf2 superfamily along with DNA methylase, it is suspected to function in the RdDM-like pathway to achieve DNA methylation in fungi (Groth et al., 2014).

### Phylogenetic Analysis of the Fungal DNMT1 Family and RID/Masc1

We surveyed the literature to identify fungal species that have both DNA methylation data and DNMT1 family and RID/Masc1 data. There were 40 such fungi, including 15 Basidiomycota species, 22 Ascomycota species, 2 Mucoromycota species, and 1 Myxomycota species (Table 2). The analysis showed that different DNMT families are present in different fungal genomes. Furthermore, there is a distinct DNMT preference between Basidiomycota and Ascomycota. Most fungal genomes in basidiomycetes contain one to three copies of DNMT1, together with only one copy of DNMT5/Rad8 families. DNMT1 and DNMT5 are the main DNMTs in Basidiomycota, which lack Dim-2 and seldom exhibit *de novo* DNA methyltransferase RID. By contrast, RID and Dim2 are the main DNMTs in Ascomycota, especially in Pezizomycotina (Bewick et al., 2019). DNMT1 is present in most eukaryotes, except for some Dikarya fungi, such as most Ascomycota (Zemach and Zilberman, 2010; Bewick et al., 2019). However, DNMTs were not observed in 5 fungi, namely, *Ustilago maydis*, *Candida albicans, Schizosaccharomyces pombe, Saccharomyces cerevisiae*, and *Yarrowia lipolytica* (Table 2; Ponger and Li, 2005; Huang et al., 2016).

**TABLE 2:** Numbers of genes encoding DNMT proteins in the 40 fungal species.

**Division**	**Subphylum**	**Order**	**Organism**	**DNA methylation**	**Dnmt1/Masc2**	**Dim-2**	**RID/Masc1**	**DNMT5**	**DNMT2**	**References**
Basidomycota	Agaricomycotina	Agaricales	*Coprinopsis cinerea*	+	2	-	-	1	1	Bewick et al., 2019
			*Laccaria bicolor*	+	2	-	-	1	1	Bewick et al., 2019
			*Agaricus bisporus*	+	2	-	-	1	1	Bewick et al., 2019
			*Schizophyllum commune*	+	2	-	-	1	1 (2 like)	Bewick et al., 2019
			*Armillaria bulbosa*	+	NA	NA	NA	NA	NA	
			*Pleurotus eryngii Subsp. Tuoliensis*	+	NA	NA	NA	NA	NA	
			*Pleurotus eryngii var. eryngii*	+	3	-	-	1	1	Zhang et al., 2018
			*Pleurotus tuoliensis*	+	3	-	-	1	1	Zhang et al., 2018
			*Pleurotus ostreatus*	+	3	-	-	1	1	Bewick et al., 2019
		Russulales	*Heterobasidion parviporum*	+	2	-	-	1	1	Zeng et al., 2019
		Polyporales	*Postia placenta*	+	2	-	-	1	1	Bewick et al., 2019
			*Ganoderma sinense*	+	2	-	-	2	1	This review
			*Sporotrichum dimorphosporum*	+	NA	NA	NA	NA	NA	
	Ustilaginomycotina	Ustilaginales	*Ustilago maydis*	+	-	-	-	-	-	Bewick et al., 2019
			*Ustilago violaceae*	+	NA	NA	NA	NA	NA	
Ascomycota	Pezizomycotina	Diaporthales	*Cryphonectria parasitica*	+	-	1	1	1	1	Bewick et al., 2019
		Eurotiales	*Aspergillus flavus*	-	-	-	1	1	-	Bewick et al., 2019
		Magnaporthales	*Magnaporthe oryzae*	+	-	1	1	-	(1 like)	Bewick et al., 2019
		Sordariales	*Neurospora crassa*	+	-	1	1	-	-	Bewick et al., 2019
			*Neurospora sitophila*	+	-	1	1	NA	NA	This review
			*Neurospora tetrasperma L1*	+	-	1	1	NA	NA	This review
			*Neurospora tetrasperma L10*	+	NA	NA	NA	NA	NA	
			*Neurospora tetrasperma L6*	+	NA	NA	NA	NA	NA	
		Hypocreales	*Cordyceps militaris*	+	-	1	1	-	-	Bewick et al., 2019
			*Fusarium oxysporum*	+	-	1	1	NA	NA	
			*Metarhizium robertsii*	+	-	1	1	-	-	Bewick et al., 2019
			*Metarhizium anisopliae*	+	-	1	1	-	-	Bewick et al., 2019
		Onygenales	*Uncinocarpus reesii*	+	-	1	1	1	-	Bewick et al., 2019
		Pezizales	*Tuber melanosporum*	+	-	1	1	1	-	Bewick et al., 2019
			*Ascobolus immersus*	+	1	1	1	1	-	Bewick et al., 2019
			*Phymatotrichum omnivorum*	+	NA	NA	NA	NA	NA	
		Pleosporales	*Cochliobolus heterostrophus*	+	-	1	1	-	-	Bewick et al., 2019
		Ophiostomatales	*Ophiostoma novo-ulmi*	+	-	1	-	-	-	Bewick et al., 2019
	Taphrinomycotina	Schizosaccharomycetales	*Schizosaccharomyces pombe*	-	-	-	-	-	1 Pmt1	Bewick et al., 2019
			*Yarrowia lipolytica*	+	-	-	-	-	-	Bewick et al., 2019
	Saccharomycotina	Saccharomycetales	*Saccharomyces cerevisiae*	-	-	-	-	-	-	Bewick et al., 2019
			*Candida albicans*	+	-	-	-	-	-	Bewick et al., 2019
Mucoromycota	Mucoromycotina	Mucorales	*Phycomyces blakesleeanus*	+	1	1	-	-	2	Bewick et al., 2019
			*Mucor rouxii*	+	NA	NA	NA	NA	NA	
Myxomycota		Physarales	*Physarum polycephalum*	+	NA	NA	NA	NA	NA	

*The numbers of each DNMT subgroup in each fungus are listed. +, detectable; -, Not-detectable; NA, Not available.*

**FIGURE 2:**
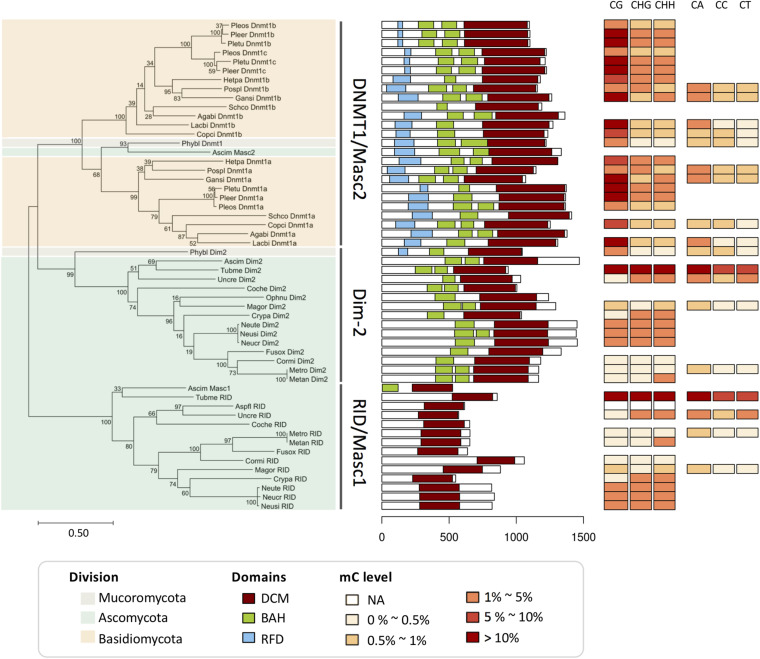
Phylogenetic and domain architecture analyses of the DNMT1 family and RID/Masc1 protein in 26 fungi. The ML tree was constructed with the conserved DCM domains of the 54 predicted proteins from 26 species using the WAG model in MEGA 7.0 with 200 bootstrap replicates. The three groups, namely, DNMT1/Masc2, Dim-2 and Masc1/RID, are indicated.

We performed a phylogenetic analysis (see Extended experimental procedures in the Supplementary Material) based on the conserved protein domains of the 54 DNMT1 family and RID/Masc1 proteins from the 26 fungal genomes (Figure 2), including 23 proteins from 10 Basidiomycota, 29 proteins from 15 Ascomycota, and 2 proteins from 1 Mucoromycota. The DNA methylation levels in the CG, CHG, CHH, CA, CC, and CT contexts of each fungal species are also shown in Figure 2. Similar to the report of Huang et al. (2016), we found that the DNMTs in these fungi were clustered into three subgroups: the DNMT1/Masc2 subgroup, Dim-2 subgroup, and RID/Masc1 subgroup. All three subgroups contained the DNA-cytosine methyltransferase (DCM) domain, while the BAH domain was absent from the RID/Masc1 subgroup. Only the DNMT1/Masc2 subgroup and *P. blakesleeanus* Dim2 contained the RFD domain, which targeted replication foci for discriminating between unmethylated and hemimethylated DNA, resulting in higher CG methylation (>0–30%) than non-CG methylation (0–5%). The DNMT1 proteins of Basidiomycota fungi specifically belonged to the DNMT1/Masc2 subgroup, not Dim-2 (Figure 2). The fungi in the Dim-2 and RID/Masc1 subgroups are from Ascomycota and Mucoromycotina but not Basidiomycota. Without the RFD domain, Dim-2 and RID/Masc1 of Ascomycota usually result in slightly higher non-CG methylation (>0–5%) than CG methylation (>0–0.5%) or equal CG and non-CG methylation (Figure 2). The phylogenetic tree also showed that Dim-2 proteins were absent in Basidiomycota, indicating that Dim-2 was lost after the bifurcation between Ascomycota and Basidiomycota (Huang et al., 2016). In contrast to the findings of Huang et al. (2016), after we blasted the DNMT1 homologs with the *Phycomyces blakesleeanus* genome, we noticed that *P. blakesleeanus* Dim2 (132318) in Huang’s research should contain BAH and RFD domains and more likely belonged to the DNMT1/Masc2 subgroup, while *P. blakesleeanus* DNMT1 (77036) in Huang’s research should belong to the Dim2 subgroup (Figure 2). Therefore, we changed the names of the two DNMTs (Supplementary Table 2).

As shown in Figure 2, the domain architecture indicated that the DNMT1-like protein sequences from different fungi were clustered into the DNMT1/Masc2, Dim-2 and Masc1/RID groups, suggesting a possible functional difference in DNA methylation among these three groups. The biological function of DNMT1 or Masc2 proteins has been demonstrated to act together with or recruit histone deacetylases in fungi (i.e., DNMT1a in *Laccaria bicolor* and *Coprinopsis cinerea*) (Fuks et al., 2000). Dim-2 has been shown to be able to catalyze DNA methylation in all sequence contexts in *N. crassa* (Kouzminova and Selker, 2001). Masc1 and RID are specific to fungi and required for *de novo* methylation associated with MIP and RIP mutations, respectively (Chen and Li, 2004; Ponger and Li, 2005; Zemach et al., 2010; Freitag, 2017).

## Comparison of DNA Methylation in Fungi

### Methods of DNA Methylation Assays

To date, several DNA methylation assays have been developed to detect cytosine methylation, including chemical analytic methods, such as high-performance liquid chromatography (HPLC), gas chromatography-mass spectrometry (GC/MS), restriction enzyme digestion of methylated DNA, sequencing and Southern blot hybridization (Table 1). Most early reports on the investigation of DNA methylation performed chemical analytic methods, such as HPLC (Fronk and Magiera, 1994). Instead of HPLC, isotope dilution GC/MS, which has a high sensitivity for analyzing 5mC directly from genomic DNA, was developed and applied in several fungal studies (Russell et al., 1987; Tang et al., 2012). In recent years, more methods have become commonly used for profiling genome-wide methylation levels, including restriction enzyme digestion of methylated DNA followed by hybridization to high-density oligonucleotide arrays or sequencing and Southern blot hybridization, methylated DNA immunoprecipitation (MeDIP) combined with large-scale analysis using microarrays for capturing methylated genomic DNA, methyl-binding domain (MBD) proteins followed by array hybridization or sequencing and WGBS (Lippman et al., 2004; Weber et al., 2005; Zhang et al., 2006; Vaughn et al., 2007). Although these approaches can determine either the status of DNA methylation or genome-wide methylation levels (i.e., HPLC and GC/MS), none of them reached single-base resolution until 2007, when WGBS based on bisulfite sequencing coupled with high-throughput sequencing was developed (Zilberman et al., 2007; Lister and Ecker, 2009; Table 1).

### WGBS, HPLC, GC/MS, and Restriction Enzyme Digestion-Based Methods Have Been Used to Profile the DNA Methylation Level of Fungi

To evaluate the resolution of methylation data generated by different methods, the 40 fungal DNA methylation levels and the assays are listed in Table 1. There were 22 fungi identified by WGBS, 12 by either HPLC or GC/MS, and 8 by restriction enzyme digestion-based methods. The methylation data of the filamentous ascomycete *N. crassa* were detected by both HPLC and WGBS, and those of *Phycomyces blakesleeanus* were detected by both restriction enzyme digestion-related methods and WGBS. We compared the DNA methylation levels of the mycelium within three phyla of fungi, namely, Mucoromycota, Ascomycota, and Basidiomycota (Table 1; Zemach et al., 2010; Montanini et al., 2014; Jeon et al., 2015; Zhu et al., 2015; Li et al., 2017). We observed high variation in DNA methylation levels, where the average 5mC% ranged from 0.39 to 12.3% when profiled by WGBS. Among these fungi, five species of Ascomycota, namely, *Magnaporthe oryzae*, *Cordyceps militaris*, *Metarhizium robertsii*, *Metarhizium anisopliae*, and *Uncinocarpus reesii*, clearly showed very low levels of DNA methylation (0.55, 0.48, 0.39, 0.60 and 0.68%, respectively). The plant-symbiotic black truffle *Tuber melanosporum* has the highest DNA methylation (12.3%), followed by the medicinal fungi *Gannoderma sinense* (4.64%), *Cryphonectria parasitica* (3.90%), and *Laccaria bicolor* (3.73%). Unlike the data detected by HPLC, GC/MS, and restriction enzyme digestion-based methods only showing the average 5mC level, the WGBS data contain both methylated CG and non-CG (CHG and CHH) sites. We compared 12 fungi that were profiled by chemical analytic methods (GC/MS and HPLC). Within the 3 profiled by GC/MS, the highest methylation level was found in *Y. lipolytica* and *N. crassa* (0.36%), followed by *C. albicans* (0.08%). The 9 fungi profiled by HPLC also showed variation in methylation ranging from 1.38 to 5.42%. Restriction enzyme digestion-related methods were used to detect the methylation levels of 8 fungi. The results show that methylation seems to occur in all of these fungi. However, more detailed information on the methylation levels was not provided due to the limitations of the assay (Table 1). Of these fungi, *Sporotrichum dimorphosporum* showed a very low methylation level (∼0.2%) in the mycelium according to restriction and nearest-neighbor analysis (RE-NNA) (Antequera et al., 1984). Fungal methylation data generated by restriction enzyme digestion-based methods should combined with those from other methods to avoid insufficient evidence for data explanation. Both *N. crassa* and *P. blakesleeanus* were subjected to two different methylation determination methods. The methylation level of *P. blakesleeanus* was 2.9% when using RE-NNA, higher than that (0.94%) detected by using WGBS (Antequera et al., 1985). On the other hand, the methylation level of *N. crassa* stationary-phase mycelia was 0.4% according to GC/MS and lower than 2.19% according to WGBS (Table 1; Hosseini et al., 2020). The results indicated that DNA methylation profiled by distinct methods was different, and it is necessary to compare the methylation data from equal baselines or the same detection method to eliminate the difference. In conclusion, among the four methods that have been reported to be used for fungal DNA methylation assays, WGBS is a better choice for obtaining more details of the fungal methylome. Therefore, in the following discussion, we will only compare fungal methylation data generated by WGBS.

### Fungal Mycelium Has the Lowest DNA Methylation Level Among Different Lifecycle Stages

**FIGURE 3:**
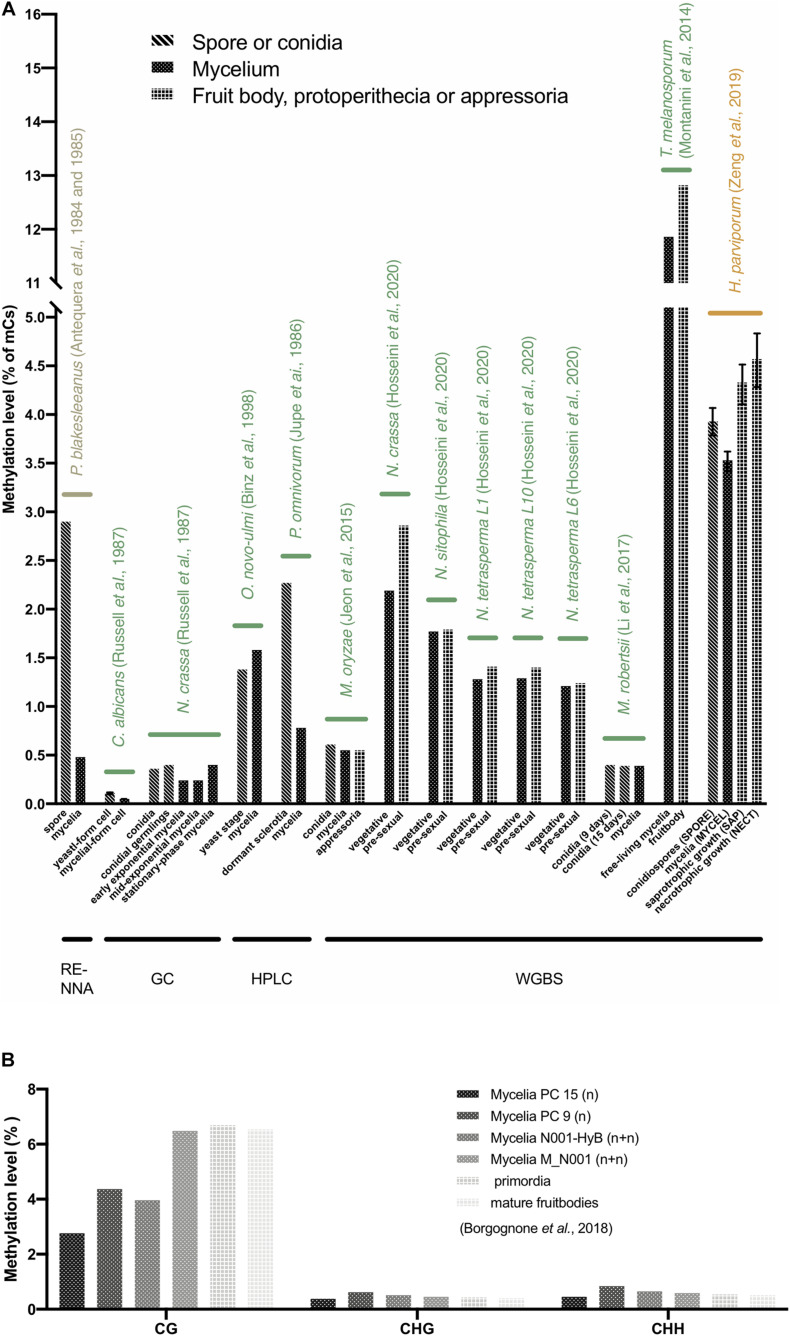
DNA methylation levels at different life stages in fungi. **(A)** The average 5mC level of different life stages in 14 fungi. The X-axis represents the different life stages of fungi. The Y-axis represents the average 5mC level. The labels with different colors indicate fungi from Mucoromycota (olive), Ascomycota (green), and Basidiomycota (yellow). The methods of DNA methylation assays are shown below. **(B)** The CG, CHG, and CHH methylation levels of *Pleurotus ostreatus* (Borgognone et al., 2018). Note that only *C. albicans* and *H. parviporum* have replicates, therefore the error bars represent the variances.

DNA methylation varied among different stages of fungal life cycles. Comparing the spore, yeast stage cell, dormant sclerotia, and conidia with mycelium of 8 fungi, namely, *P. blakesleeanus*, *C. albicans*, *Ophiostoma novo-ulmi*, *Phymatotrichum omnivorum*, *M. oryzae, N. crassa*, *M. robertsii*, and *Heterobasidion parviporum*, showed that regardless of which method was used for the DNA methylation assay, the lowest methylation levels were mostly found in the mycelium stage, and a higher methylation level was found in the spore or conidia stage of these fungi, except for *O. novo-ulmi* (Figure 3A). The lower methylation level was also observed in the mycelium stage compared to the fruit body formation stage and the other infection phases, such as protoperithecia of 5 *Neurospora* species and strains, appressoria of *M. oryzae*, saprotrophic and necrotrophic growth of *H. parviporum*, and fruitbodies of *T. melanosporum* (Figure 3A). In *Pleurotus ostreatus*, the monokaryotic mycelium stage had the lowest CG methylation level, while the two strains showed differences. In contrast, the non-CG (CHG, CHH) methylation level of mycelium was slightly higher than that of primordia and mature fruitbodies, although non-CG contexts were not predominant in *P. ostreatus* (Figure 3B; Borgognone et al., 2018). Additionally, the methylation level of the exponentially growing mycelia of *N. crassa* was lower than that of the stationary-phase mycelia (Figure 3A) (Russell et al., 1987). A lower methylation level may increase genome plasticity and the ability to adapt to environmental changes. Therefore, a lower methylation level in the mycelium stage may allow the genome to undergo evolution and avoid silencing of growth and development processes, especially in monokaryons, the most commonly found developmental stage with high potential in nature (Antequera et al., 1984; Antequera et al., 1985; Russell et al., 1987; Montanini et al., 2014; Borgognone et al., 2018).

### DNA Methylation Preferentially Occurs in TE and Repeat Regions in Fungi

DNA methylation can be found in widespread genomic regions, such as gene bodies, promoters, and TEs, causing the DNA methylation pattern to vary across eukaryotes (Jeltsch, 2010; Law and Jacobsen, 2010; Su et al., 2011). For example, both CG and non-CG methylation of plants occurred in the promoters of silent genes and TEs. However, in the transcribed genes of animals and plants, DNA methylation was not found around their promoters but within the gene bodies, except in the TSS-proximal regions. Additionally, TEs are also the targets of methylation in plants and some animals (Taiko et al., 2015; de Mendoza et al., 2020).

In contrast to plants and animals, most fungi lack gene body methylation. In contrast, DNA methylation was enriched in TEs and repeats in the fungal genome (Bewick et al., 2019). As shown in Table 1, the methylation of 12 fungi, namely, *Coprinopsis cinerea*, *L. bicolor*, *P. placenta, G. sinense, P. eryngii var. eryngii, P. tuoliensis, P. ostreatus, H. parviporum, T. melanosporum*, *M. oryzae*, *Neurospora*, and *P. blakesleeanus*, mainly occurred in TE and repeat regions rather than gene bodies (Zemach et al., 2010; Montanini et al., 2014; Jeon et al., 2015; Zhu et al., 2015; Borgognone et al., 2018; Zhang et al., 2018; Zeng et al., 2019; Hosseini et al., 2020). The result of Figure 5 also showed that in all of the three sequence contexts (CG, CHG, and CHH) of the nine fungi, TEs showed the highest methylation level, followed by intergenic regions, comparing to the genebodies and exons that showed less methylation. In the *T. melanosporum*, TEs were methylated at approximately 80% of CpG sites, whereas genes had almost no methylation, suggesting a non-exhaustive and partly reversible methylation process. This process would lead to transcriptional activation of TEs, thereby potentially playing a role in promoting genome plasticity and the environmental fitness of *T. melanosporum* (Montanini et al., 2014). In *Neurospora*, both transcriptional and posttranscriptional gene silencing mechanisms are used for TE silencing, including the RID involved in RIP mutation (Aramayo and Selker, 2013; Hosseini et al., 2020). For the model plant pathogenic fungus *M. oryzae*, DNA methylation occurs in and around genes as well as TE regions and undergoes global reprogramming during fungal development (Jeon et al., 2015). In contrast to those in previous fungi, the methylation patterns in *U. reesii* and an entomopathogenic fungus, *M. robertsii*, showed clear gene body methylation (Li et al., 2017).

DNA methylation of fungi affects gene expression by methylating surrounding TEs instead of directly methylating the gene bodies and their promoters. In *Pleurotus ostreatus* and *Pleurotus tuoliensis*, the genes near TEs had higher methylation levels and lower gene expression than those far away from TEs (Borgognone et al., 2018; Zhang et al., 2018). Additionally, the genes located inside TE-rich clusters also had higher methylation levels and lower gene expression (Borgognone et al., 2018). Genes of *L. bicolor* with TE insertions within a 1 kb upstream and downstream window were repressed. Nevertheless, in *S. cerevisiae*, a fungus lacking DNA methylation, the genes under TE influence did not show any alteration in expression (Castanera et al., 2016). As a result, TE-associated methylation affected the expression of surrounding genes.

### Two Distinct DNA Methylation Patterns, CG and Non-CG Preference, Are Found in Fungi and Associated With DNA Methyltransferase Combinations

**FIGURE 4:**
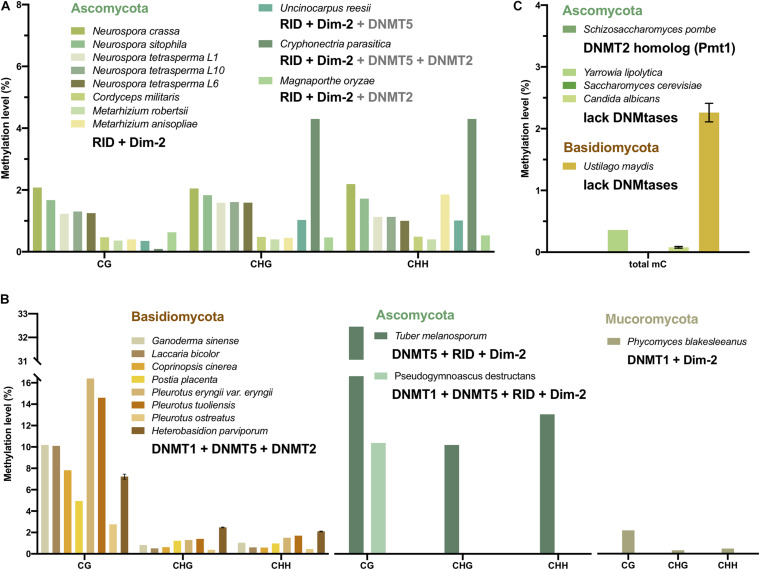
CG, CHG, and CHH methylation levels and the DNMT combination of fungi. The methylation level values are from mycelium, not the other stages. The bars with different colors represent fungi from Mucoromycota (olive), Ascomycota (green), and Basidiomycota (yellow). **(A)** The DNA methylation level and the DNMT combination of fungi showed a preference for CHG and CHH methylation. **(B)** The DNA methylation level and the DNMT combination of fungi showed a preference for CG methylation. **(C)** The DNA methylation level of fungi without DNMT or with only tRNA methyltransferase. Note that only *C. albicans*, *U. maydis*, and *H. parviporum* have replicates, therefore the error bars represent the variances.

In mammals, CG methylation is predominant. Some of the methylation patterns in plants are similar to those in mammals, except for the two non-CG methylation systems specific to CHG and CHH. These two systems are also present in fungi (Jeltsch, 2010). Moreover, two distinct methylation patterns were found in fungi. One showed a strong preference for CG methylation, and the other showed a preference for CHG and CHH methylation (Figure 4 and Table 1).

**FIGURE 5:**
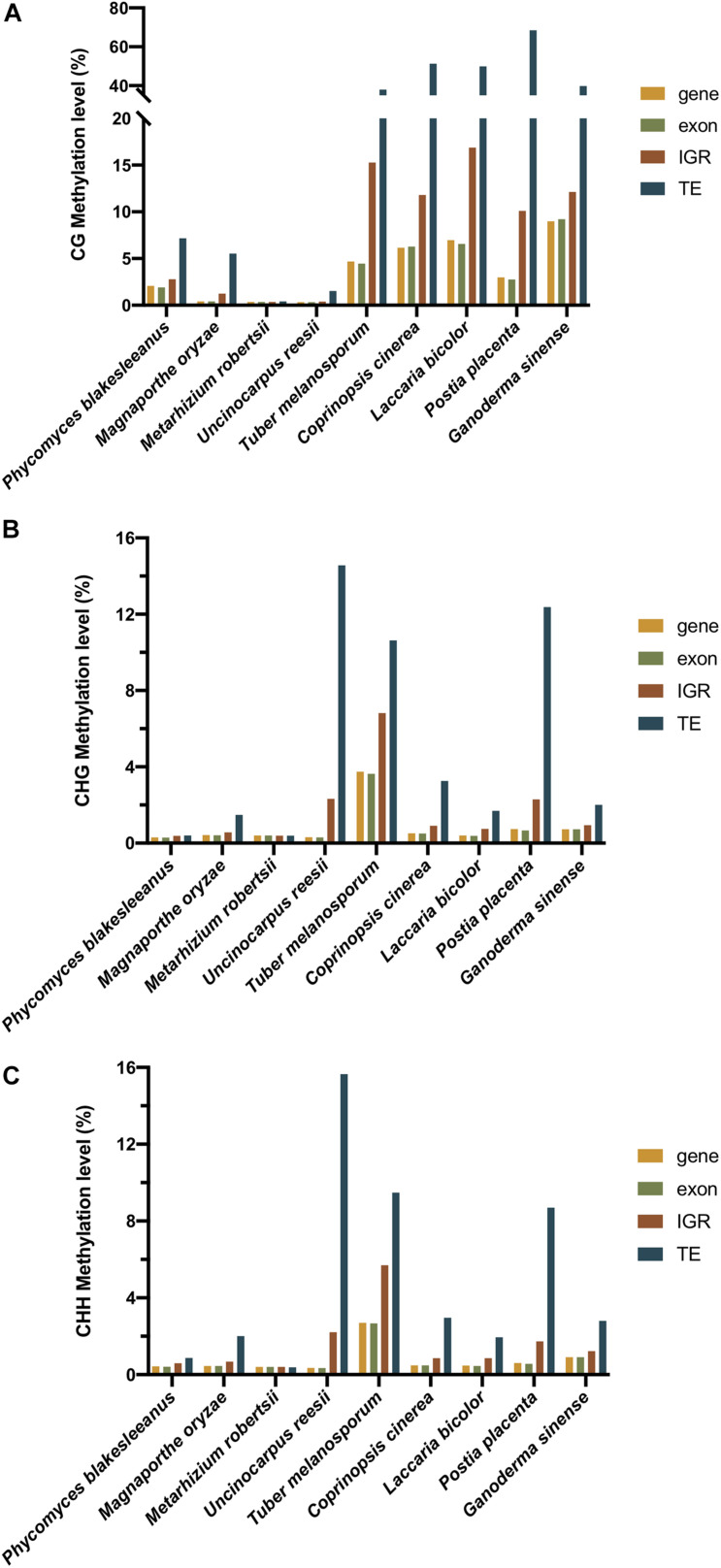
The **(A)** CG, **(B)** CHG, and **(C)** CHH methylation level of different regions of nine fungi (*C. cinerea*, *L. bicolor*, *P. placenta*, *U. reesi*, *G. sinense*, *M. oryzae*, *M. robertsii*, *T. melanosporum*, and *P. blakesleeanus*). The yellow, green, red, and blue bars represent the methylation level of genebody, exon, intergenic region (IGR), and transposable element (TE), respectively.

The DNA methylation of 11 Ascomycota, including 5 *Neurospora* species and strains, *C. militaris*, *M. robertsii, M. anisopliae*, *U. reesii*, *C. parasitica*, and *M. oryzae*, had no preference for CG sites. The methylation level of the non-CG context, which was 0.4–4.3%, was generally similar to and even higher than that of the CG context (0.09–2.08%) (Figure 4A and Table 1). The main DNA methyltransferase combination of these fungi was RID + Dim-2, with some additional DNMT5 (Table 2). CG and non-CG sites might be equally methylated by RID/Masc1 and Dim-2, which are responsible for both symmetric and asymmetric site methylation and are required for *de novo* DNA methylation and the maintenance of DNA methylation, respectively. In *U. reesii*, the corresponding increase in CA and TG dinucleotides was consistent with the RID in the genome and involved RIP mutation (Galagan and Selker, 2004).

By contrast, the DNA methylation of 8 Basidiomycota, including *G. sinense, L. bicolor*, *C. cinerea*, *P. eryngii var. eryngii, P. tuoliensis, P. ostreatus*, and *H. parviporum*, revealed substantial CG methylation (2.76–16.4%) and a small amount of non-CG methylation (0.38–2.47%) (Figure 4B and Table 1). DNMT1 and DNMT5, acting as CG site maintenance enzymes, were the main DNMTs in Basidiomycota (Table 2). In addition, *T. melanosporum* and *Pseudogymnoascus destructans* of Ascomycota also showed significantly high CG methylation and low non-CG methylation, while DNMT1 or DNMT5 as well as RID and Dim-2 was found in these two fungi (Figure 4B). The CG methylation of *P. blakesleeanus* of Mucoromycota with DNMT1 and Dim-2 was also higher than the non-CG methylation (Figure 4B). Therefore, the DNA methylation pattern in fungi was correlated with the DNA methyltransferase combination and associated with the classification of different phyla of fungi.

### Fungal Species With an Absence of DNA Methylation Lack DNMTs

DNA methylation was not found in several fungal species; more than 20 yeast strains were described as having no detectable DNA methylation levels by GC/MS or LC-MS/MS (Tang et al., 2012; Capuano et al., 2014). *Aspergillus flavus*, *Schizosaccharomyces pombe* and *Saccharomyces cerevisiae* have non-detectable methylation based on WGBS, LC-MS/MS and HPLC methods (Binz et al., 1998; Liu et al., 2012; Capuano et al., 2014). A lack of DNMT homologous in the *Saccharomyces cerevisiae* genome was reported (Huang et al., 2016; Figure 4C), which can lead to no DNA methylation. *S. pombe* has a homolog of the DNMT2 family, termed Pombe Methyl Transferase 1 (Pmt1). However, Pmt1 appeared to be specific to tRNA modification and not associated with DNA methylation (Table 2; Wilkinson et al., 1995; Becker et al., 2012). *A. flavus* has one DNMT1 and one DNMT5, and the methylation level of *A. flavus* is quite low (similar to that of unmethylated lambda DNA, 0.44∼0.47%); hence, DNA methylation is considered absent in *A. flavus*, or *de novo* DNA methylation occurs transiently during the obscure sexual stage (Liu et al., 2012). Nonetheless, there is still low methylation in a few fungi lacking DNMTs, such as *Y. lipolytica* (0.36%) and *Ustilago maydis* (2.26%) (Figure 4C).

### The Evidence for DNA Methylation Involvement in Fungal Life Stages

Variable DNA methylation levels are found in fungi and their life stages, thereby leaving their biological role an open question, especially in their life cycles. Fungal genomic studies that describe DNA methylation levels and methylation preference sites are still limited. To date, more than 80 fungi have been subjected to whole-genome sequencing. Of these, only 40 fungi have methylation data available, and 14 fungi have descriptions of methylation in different life stages (Figure 3A). Different DNA methylation levels were found in different life stages in 14 fungi; lower methylation levels were usually observed in mycelium (Figure 3A).

*P. blakesleeanus* showed 0.48 and 2.9% methylation levels in mycelium and spores, respectively, suggesting that gene expression may be regulated by DNA methylation and thereby impact the different stages of the life cycle (Figure 3A). Indeed, in *P. blakesleeanus*, it has been shown that methylation can repress loci transcriptionally; thus, active genes are often unmethylated (Antequera et al., 1985). A higher methylation level was found in the sclerotia of *P. omnivorum* than in the mycelium (Figure 3A). In *P. omnivorum*, the sclerotium is the structure for adapting to adverse conditions and allows survival for more than a decade deep in the soil. Therefore, this structure is a potential source of inactive genes and shows a high methylation level (Jupe et al., 1986). The DNA methylation level in gene-flanking regions of the forest pathogen *H. parviporum* with dual life strategies, saprotrophy on dead wood and necrotrophy on living trees, changes between different stages, suggesting that DNA methylation may contribute to condition-specific gene expression.

At different life stages of fungi, variable methylation may imply complicated epigenetic regulation, possibly associated with changes in morphology upon life stage switches. In *T. melanosporum*, hypomethylated or unmethylated TEs were found to be transcriptionally active, with higher expression levels in free-living mycelium (FLM) compared to fruitbodies (FBs) (Montanini et al., 2014).

In Basidiomycota, *C. cinerea* is a model species in studies of photomorphogenesis; it has been revealed that the fruiting mechanism requires specific environmental conditions, such as temperatures between 25°C and 28°C, humidity >85% and a typical day/night rhythm, implying epigenetic coregulation (Kamada et al., 2010). However, the life stage switch does not seem to be conserved among Basidiomycota fungi.

## Discussion and Perspectives

In 2010, keystone research on summarized that genome-wide DNA methylation was an important process in evolutionary adaptation and conservation (Jeltsch, 2010; Zemach et al., 2010; Su et al., 2011). In this review, we provided an overview of fungal DNA methyltransferases, as well as the recent reports on the potential association between fungal DNMT5 and the RdDM pathway. Furthermore, we collected DNA methylation data (reported statistics and raw sequencing data whichever available) and domain features of all the DNA methyltransferases from 40 fungal species to present a comprehensive summary of current fungal DNA methylation, pointing to several directions and gaps for future investigation.

As summarized in our review, the correlation amongst the fungal DNA methylation levels, phylogenies of the fungal DNMT1 family and RID/Masc1 domains, and fungal DNMTs combinations showed that the patterns of DNA methylation are closely associated with the DNMTs. Since the fungal taxonomy has undergone major challenges in the past few decades according to the rapid development in fungal genomics, the highly-diverse features of the species such as morphologies, habitats, lifestyles and epigenomes together with the genetic data may present a comprehensive profile for individual fungal species, to provide an improved description for fungal diversity.

Different fungi often have different types of DNMTs in their genomes, leading to high divergence in their methylation profiles. In truffle, treatment of 5-azacytidine, an inhibitor of DNA methyltransferases, is used to explore the correlation between fungal phenotypes and the potential epigenetic regulation by DNA methylation (Montanini et al., 2014). Still, the epigenetic regulation in fungi is still largely unknown. In addition to DNA methylation, histone modifications and sRNA are also reported to play an important role on diverse aspect of fungal biology (Brosch et al., 2008; Nakayashiki and Nguyen, 2008; Smith et al., 2010; Chang et al., 2012; Jeon et al., 2014; Honda et al., 2016; Dubey and Jeon, 2017; Freitag, 2017; Elías-Villalobos et al., 2019; Chung et al., 2020). There are growing observations describing the profiles of different kinds of epigenetic marks in fungi, yet the studies of integrated fungal epigenetic modifications are limited in only a handful of fungal species, such as *N. crassa*, *S. pombe*, and *S. cerevisiae*. In *Neurospora crassa*, DNA methylation requires histone deacetylases (HDACs). The mutation of histone deacetylase genes causes global increase in histone acetylation level in correlation with the site-specific loss of DNA methylation (Smith et al., 2010); Trichostatin A (TSA), the inhibitor of HDAC activity, is found to result in the selective loss of demethylation (Selker, 1998). In addition, the histone deacetylase complex HCHC works with the DNMT complex DIM-2–HP1 to establish and maintain heterochromatin, is required for proper DNA methylation of *N. crassa* (Honda et al., 2012, 2016). Altogether the observations between DNA methylation and HDACs activities suggest a close association between DNA methylation and histone modification in fungal epigenomes.

As fungi tend to have close interactions with the plants, animals and the environments, complicated epigenomic regulations may be involved. By combining the epigenome data of DNA methylation and other epigenetic marks will definitely warrant future fungal research.

## Author Contributions

Y-SN and Y-CH drafted the manuscript. M-RY performed phylogenetic analysis and methylome reanalysis. P-YC coordinated the study and edited the manuscript. All authors read and approved the final manuscript.

## Conflict of Interest

The authors declare that the research was conducted in the absence of any commercial or financial relationships that could be construed as a potential conflict of interest.
