# Supplementary material for: Diversity of Fungal DNA Methyltransferases and Their Association With DNA Methylation Patterns
Source: Front Microbiol. 2021 Jan 22;11:616922. doi: 10.3389/fmicb.2020.616922 (PMC7862722; doi:10.3389/fmicb.2020.616922)
Supplement: Supplementary file 3 [file Data_Sheet_3.docx]

**Supplementary Material**

Diversity of fungal DNA methyltransferases and their association with DNA methylation patterns

Yu-Shin Nai, Yu-Chun Huang, Ming-Ren Yen, and Pao-Yang Chen

**EXTENDED EXPERIMENTAL PROCEDURES**

**Phylogenetic analysis of fungal DNMT1**

Phylogenetic analysis was performed based on the conserved protein domains of the 54 DNMT1 family and RID/Masc1 proteins from the 26 fungal genomes (Supplementary Table 1). The amino acid sequences of conserved protein domains were aligned using the Conserved Domain Database (CDD) with default parameters [1, 2]. The phylogenetic tree was build based on the WAG model [3] with the maximum likelihood (ML) method implemented in MEGA X [4]. The consensus trees were deducted from 200 bootstrap replicates for ML analysis.

**Reanalysis of Bisulfite Sequencing data**

Nine whole-genome bisulfite sequencing (WGBS) fungal datasets were reanalyzed according to Montanini *et al*., 2014 [5]. Reads from each set of WGBS data (Supplementary Table 2) were aligned to the reference genome (Supplementary Table 2) using BS Seeker [6]. Genome-wide DNA methylation profiles were generated by determining methylation levels for each cytosine in the genome. Since bisulfite treatment converts unmethylated cytosines (Cs) to thymines (Ts) after PCR amplification, the methylation level at each cytosine was estimated as #C/(#C + #T), where #C is the number of methylated reads and #T is the number of unmethylated reads. The methylation level per cytosine serves as an estimate of the percentage of cells bearing a methylated cytosine at a specific locus. We only included cytosines that were covered by at least four loci.

**Supplemental references**

1. Marchler-Bauer, A., et al., *CDD: NCBI's conserved domain database.* 2015. **43**(D1): p. D222-D226.

2. Lu, S., et al., *CDD/SPARCLE: the conserved domain database in 2020.* 2020. **48**(D1): p. D265-D268.

3. Whelan, S., N.J.M.b. Goldman, and evolution, *A general empirical model of protein evolution derived from multiple protein families using a maximum-likelihood approach.* 2001. **18**(5): p. 691-699.

4. Kumar, S., et al., *MEGA X: molecular evolutionary genetics analysis across computing platforms.* 2018. **35**(6): p. 1547-1549.

5. Montanini, B., et al., *Non-exhaustive DNA methylation-mediated transposon silencing in the black truffle genome, a complex fungal genome with massive repeat element content.* Genome Biol, 2014. **15**(7): p. 411.

6. Chen, P.Y., S.J. Cokus, and M. Pellegrini, *BS Seeker: precise mapping for bisulfite sequencing.* BMC Bioinformatics, 2010. **11**: p. 203.
