# Supplementary figures and images for: Diversity of Fungal DNA Methyltransferases and Their Association With DNA Methylation Patterns
Source: Front Microbiol. 2021 Jan 22;11:616922. doi: 10.3389/fmicb.2020.616922 (PMC7862722; doi:10.3389/fmicb.2020.616922)

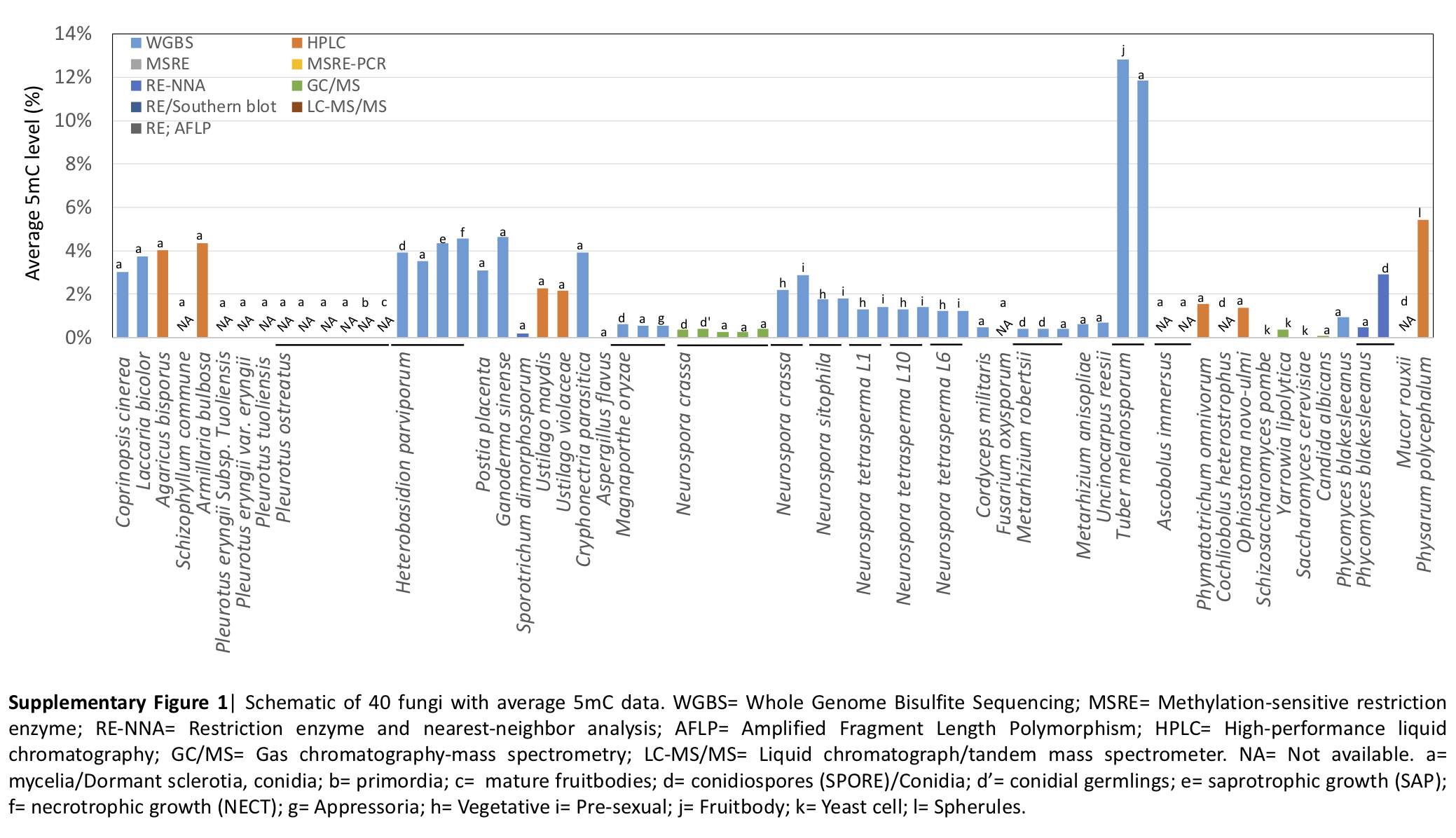

Supplement: Supplementary file 4 [file Image_1.tiff]
